# Correction to: Chromosome-level genome assembly of the common chiton, *Liolophura japonica* (Lischke, 1873)

**DOI:** 10.46471/gigabyte.159

**Published:** 2025-09-09

**Authors:** Hong Kong Biodiversity Genomics Consortium, Jerome H. L. Hui, Ting Fung Chan, Leo Lai Chan, Siu Gin Cheung, Chi Chiu Cheang, James Kar-Hei Fang, Juan Diego Gaitan-Espitia, Stanley Chun Kwan Lau, Yik Hei Sung, Chris Kong Chu Wong, Kevin Yuk-Lap Yip, Yingying Wei, Ming Fung Franco Au, Wai Lok So, Wenyan Nong, Tin Yan Hui, Brian Kai Hin Leung, Gray A. Williams

**Affiliations:** ^1^ School of Life Sciences, Simon F.S. Li Marine Science Laboratory, State Key Laboratory of Agrobiotechnology, Institute of Environment, Energy and Sustainability, The Chinese University of Hong Kong, Hong Kong, China; ^2^ School of Life Sciences, State Key Laboratory of Agrobiotechnology, The Chinese University of Hong Kong, Hong Kong SAR, China; ^3^ State Key Laboratory of Marine Pollution and Department of Biomedical Sciences, City University of Hong Kong, Hong Kong SAR, China; ^4^ State Key Laboratory of Marine Pollution and Department of Chemistry, City University of Hong Kong, Hong Kong SAR, China; ^5^ Department of Science and Environmental Studies, The Education University of Hong Kong, Hong Kong SAR, China; ^6^ State Key Laboratory of Marine Pollution, Research Institute for Future Food, and Department of Food Science and Nutrition, The Hong Kong Polytechnic University, Hong Kong SAR, China; ^7^ The Swire Institute of Marine Science and School of Biological Sciences, The University of Hong Kong, Hong Kong SAR, China; ^8^ Department of Ocean Science, The Hong Kong University of Science and Technology, Hong Kong SAR, China; ^9^ Science Unit, Lingnan University, Hong Kong SAR, China; ^10^ School of Allied Health Sciences, University of Suffolk, Ipswich, IP4 1QJ, UK; ^11^ Croucher Institute for Environmental Sciences, and Department of Biology, Hong Kong Baptist University, Hong Kong SAR, China; ^12^ Department of Computer Science and Engineering, The Chinese University of Hong Kong, Hong Kong SAR, China; ^13^ Sanford Burnham Prebys Medical Discovery Institute, La Jolla, CA, USA; ^14^ Department of Statistics, The Chinese University of Hong Kong, Hong Kong SAR, China; ^15^ The Swire Institute of Marine Science and School of Biological Sciences, The University of Hong Kong, Hong Kong SAR, China

This is a correction to: Hong Kong Biodiversity Genomics Consortium. Chromosome-level genome assembly of the common chiton, *Liolophura japonica* (Lischke, 1873). *GigaByte*, 2024 Apr 25; **2024**: gigabyte123. https://doi.org/10.46471/gigabyte.123.

In the originally published version of this manuscript we believed the species sequenced was *Liolophura japonica* (Lischke, 1873) [1]. Based on a recent publication analysing the molecular phylogeny of molluscs [2], we agree with the authors that the species of our genome resource should be *Liolophura sinensis* instead of *L. japonica*.

Where any reference is made to *L. japonica* this should instead be *L. sinensis*. The metadata of the supported data in NCBI has also been updated, as has the title of the data in figshare [3].
